# Estimating the Magnitude of Genetic Factors by Calculating the Genetic Relative Risk of Stroke in First-Ever Lacunar Stroke Patients

**DOI:** 10.1371/journal.pone.0021439

**Published:** 2011-06-29

**Authors:** Iris L. H. Knottnerus, Marij Gielen, Jan Lodder, Rob P. W. Rouhl, Julie Staals, Robert Vlietinck, Robert J. van Oostenbrugge

**Affiliations:** 1 Department of Neurology, Maastricht University Medical Centre, Maastricht, The Netherlands; 2 Cardiovascular Research Institute Maastricht (CARIM), Maastricht University Medical Centre, Maastricht, The Netherlands; 3 Department of Neurology, Medisch Spectrum Twente, Enschede, The Netherlands; 4 Section of Complex Genetics, Department of Genetics and Cell Biology, Nutrition and Toxicology Research Institute Maastricht (NUTRIM), Maastricht, The Netherlands; 5 Unit of Genetic Epidemiology, Department of Public Health, Epidemiology and Biostatistics, University of Birmingham, Birmingham, United Kingdom; 6 Department of Human Genetics, Faculty of Medicine, Catholic University of Leuven, Leuven, Belgium; Innsbruck Medical University, Austria

## Abstract

**Background:**

Positive family history of stroke is an independent risk factor for lacunar stroke. However, the magnitude of familial aggregation of a certain disease is better evaluated by the genetic relative risk. This is calculated by dividing the prevalence of specific disease in family members of patients by the prevalence of this disease in the general population. In a cohort of lacunar stroke patients, who were subtyped clinically and radiologically, we determined the genetic relative risk of stroke.

**Methods:**

By questionnaire and additional interview, we obtained a complete first-degree family history of stroke. The prevalence of stroke in first-degree relatives of these lacunar stroke patients was compared to the self-reported prevalence of stroke in a Dutch community based cohort of elderly volunteers. Secondly, the influence of proband characteristics and family composition on parental and sibling history of stroke were evaluated.

**Principal Findings:**

We collected data of 1066 first-degree relatives of 195 lacunar stroke patients. Strokes occurred in 13.5% of first-degree relatives. The genetic relative risk was 2.94 (95%CI 2.45–3.53) for overall first-degree relatives, 4.52 (95%CI 3.61–5.65) for patients' parents and 2.10 (95%CI 1.63–2.69) for patients' siblings. Age of proband and proband status for hypertension influenced the chance of having a parent with a history of stroke whereas the likelihood of having a concordant sibling increased with sibship size.

**Conclusions:**

We found an increased genetic relative risk of stroke in first-degree relatives of patients with lacunar stroke. Our data warrant further genomic research in this well-defined high risk population for stroke.

## Introduction

Up till now, studies on genetic epidemiology of stroke consisted mostly of family history (FH) studies, which defined positive FH of stroke as the presence of at least one affected first-degree relative (FDR). These studies found an association between FH of stroke and lacunar stroke, with odds ratios varying from 1.79 to 2.76.[1], [2], [3] As the chance of the presence of a common disease in a family increases with family size, a better approach to evaluate the magnitude of the contribution of genetic factors in a certain disease is to estimate the genetic relative risk (GRR), also called recurrence risk ratio (λ_R_). The GRR is calculated by dividing the (life-time) prevalence of a certain disease in family members of patients by its prevalence in the general population. A higher prevalence of disease in family members of patients compared to the general population, expressed by an elevated (>1) GRR, corresponds to the involvement of genes in the disease. For many complex diseases, the average GRR in FDR is around 2.[4] For stroke, a sibling recurrence risk ratio (λ_s_) of 1.66 was reported [5].

We confined our study to patients with well defined lacunar stroke. As stroke is a heterogeneous disease, the abovementioned figures might differ between stroke subtypes. In other words, the contribution of genetic factors might differ between stroke subtypes, as different pathophysiological mechanisms underlie stroke-subtypes, i.e. cardio-embolic stroke versus small artery disease. Also, several conventional vascular risk factors are involved in the aetiology of stroke and these factors themselves also have a substantial genetic component.

The aim of the present study was to determine the GRR of stroke in FDR of a cohort first-ever lacunar stroke patients who were subtyped clinically and radiologically. We designed this study as an exploration to gain insight in the eligibility of lacunar stroke patients for further genomic research. Secondly, the influences of the conventional vascular risk factors and sibship size were evaluated.

## Materials and Methods

### Participants

The consecutive registration of residential stroke patients from May 2003 until December 2007 at the Maastricht University Medical Centre (MUMC) was described earlier.[6] From this registry lacunar stroke patients were included if they had a first-ever lacunar stroke, which was defined as (1) one of the recognized lacunar syndromes with a lesion on imaging compatible with the occlusion of a single perforating artery or if no such lesion was visible on imaging, (2) established criteria of unilateral motor and/or sensory signs that involved the whole of at least 2 of the 3 body parts (face, arm and leg) without disturbance of consciousness or cortical functions were used.[7] To increase likelihood that the lacunar syndrome had resulted from small vessel disease, we excluded patients with a potential cardioembolic source of the embolus (mainly atrial fibrillation) and those with severe precerebral large vessel disease (at least one internal carotid artery with more than 50% stenosis). Furthermore, if a monogenic cause of cerebral small vessel disease (e.g. CADASIL) was considered, specific genetic tests were applied to confirm the diagnosis and those patients were not included. By applying the same criteria, we also recruited 25 lacunar stroke patients from a nearby hospital (Orbis Medical Center, Sittard, The Netherlands) and retrospectively 60 patients from the earlier stroke registry in the MUMC (February 1999-September 2002). The final sample included 195 patients with first-ever lacunar stroke. Over 95% of the patients were of native Dutch Origin. Clinical characteristics were documented at time of stroke as reported previously: age, gender, hypertension, diabetes mellitus, previous or current smoking, and coronary artery disease.[6]

### Brain imaging

MR-images (axial T2-weighted fast spin echo and fluid attenuated inversion recovery) in 157 cases and CT-images in 38 cases were obtained with a median of 22 days (interquartile range 4–69 days) after the ischemic event. Two experienced vascular neurologists (JL and RJvO) assessed the images by consensus. The inter-observer agreement, expressed by Cohen's kappa (κ), was determined prior to this study and was 0.89 for symptomatic lacunar infarct.[8]

### Obtaining FH of stroke in patients and general population

A first-degree FH of stroke was obtained by a written questionnaire given to the patient. The patient received an oral instruction containing the question “Did one of your parents or siblings ever suffer from a stroke, diagnosed by a physician?”. During regular follow up visits to the outpatient clinic, information was checked by one of the vascular neurologists (JL and RJvO) or residents (ILHK, RPWR and JS) by questions on clinical picture in family-members. If patients were not able to visit the outpatient clinic, information was checked with patient or close relative by telephone by the one of the residents (ILHK). As such, we collected ‘self’-reported data, not ‘doctor-confirmed’ data.

Information on the self-reported prevalence of stroke in the general population was extracted from the Dutch Rotterdam study.[9] In this study self-reported prevalence of stroke was obtained by a similar question as in our study. Complete data of the Rotterdam study were available for 7661 individuals aged 55 years and older, of which 352 (4.5%) reported a stroke (152 of 3034 men and 200 of 4627 women). The mean age was 69.9 years for men and 71.8 years for women.[9]

Finally, forty-nine patients visiting the outpatient neurology clinic of the MUMC for non-cerebrovascular disease and free of hypertension, diabetes mellitus and clinically evident cardiovascular disease, participated as healthy controls. These persons received the questionnaire and oral instruction, as described above. These data were used to calculate the GRR in our general population compared to the general population of Rotterdam.

### Ethics

The study protocol was approved by local research ethics committees of both hospitals (Maastricht University Medical Centre and Orbis Medical Centre, Sittard), and written informed consent was obtained from all participants.

### Statistical analysis

Data are presented as mean ± standard deviation (SD), as all continuous variables were normally distributed. Categorical variables are presented as frequencies (%).

We calculated the GRR by dividing the prevalence of stroke in known family members of lacunar stroke patients by the prevalence of stroke in the Rotterdam study. The GRR was calculated for all FDR, male FDR, female FDR, parents, mothers, fathers, siblings, sisters and brothers.

By binary logistic regression analysis we evaluated the relationship of proband characteristics (age at time of stroke, gender and vascular risk factors) to a dichotomized FH of stroke in respectively parents and siblings. As the number of known siblings might influences the chance of having a positive family history of stroke, the relationship between proband characteristics and sibling family history of stroke, was adjusted for sibship size in a second model.

## Results

The mean age of our cohort of 195 first-ever lacunar stroke patients was 63.1±11.6 years and 60% was of male gender. Hypertension, diabetes mellitus and smoking were present in respectively 60%, 14% and 53% of patients (table 1).

**Table 1:** Clinical characteristics of 195 lacunar stroke patients.

Clinical characteristics of probands	Mean ± SD or number (%)
Age (in years)	63.1±11.6
Male Gender	117 (60)
Hypertension	117 (60)
Diabetes mellitus	27 (14)
Current or previous smoking	103 (53)
Coronary artery disease	26 (13)

Data are presented as mean ± SD or number of affected patients (%).

Information on the prevalence of stroke could be obtained of 1066 FDR, consisting of 371 parents and 695 siblings. As most parents had died at time of our study, reasonable information on mean age of parents cannot be given. Respectively 6 (3%), 64 (33%) and 125 (64%) of probands had both, one or none parent with a history of stroke. The mean age of siblings was 62.9±13.5 years (62.9±13.7 for brothers and 62.8±13.2 for sisters) and the median number of siblings was 3 (IQR 2–5). In 23% of probands at least one sibling had a history of stroke and 88% of probands had at least one non-affected (discordant) sibling (table 2).

**Table 2:** Composition of families of 195 lacunar stroke patients.

Composition of families		Number (%)	
**Parents**			
	History of stroke	None	125 (64)
		One	64 (33)
		Both	6 (3)
**Siblings**			
	History of stroke	None	150 (77)
		One	31 (16)
		Two or more	14 (7)
	No history of stroke	None	23 (12)
		One	27 (14)
		Two	45 (23)
		Three or more	100 (51)

Data are presented as number of patients (%). For example, 125 patients had no parent with a history of stroke.

A total of 144 of 1066 FDR (13.5%) reported a stroke, from which a GRR for FDR of lacunar stroke patients of 2.94 (95%CI 2.45–3.53) could be calculated. Regarding parents, 77 of 371 (20.8%), suffered a stroke, resulting in a GRR of 4.52 (95%CI 3.61–5.65), whereas 67 of 695 (9.6%) of siblings had a history of stroke, leading to a GRR of 2.1 (95%CI 1.63–2.69). Thirteen of 292 FDR of 49 healthy controls reported a stroke (4.5%), resulting in a GRR for FDR of healthy controls of 0.97 (95%CI 0.56–1.66) (table 3).

**Table 3:** Genetic Relative Risk (GRR) compared to general population of Rotterdam (see text).

		Number known	Number affected (%)	Genetic Relative Risk
**Lacunar stroke patients (n = 195)**				
**FDR**	All	1066	144 (13.5)	2.94 (2.45–3.53)
	Female	519	74 (14.3)	3.30 (2.51–4.14)
	Male	547	70 (12.8)	2.55 (2.14–3.62)
**Parents**	All	371	77 (20.8)	4.52 (3.61–5.65)
	Mother	186	45 (24.2)	5.60 (4.20–7.47)
	Father	185	32 (17.3)	3.45 (2.43–4.91)
**Sibs**	All	695	67 (9.6)	2.10 (1.63–2.69)
	Sister	333	29 (8.7)	2.01 (1.39–2.93)
	Brother	362	38 (10.5)	2.10 (1.49–2.94)
**Healthy controls (n = 49)**				
	All FDR	292	13 (4.5)	0.97 (0.56–1.66)

Figures are numbers of known and affected family-members (%) and genetic relative risk (with 95% CI). FDR indicates first-degree relatives.

There was a decrease in the likelihood of having at least one concordant parent with increasing age of proband (OR 0.97 [95%CI 0.95–1.00] per year). The likelihood of having an concordant parent, but not sibling, increased with proband status of hypertension (OR 2.28 [95%CI 1.12–4.32]). Neither proband gender nor proband status for smoking, diabetes mellitus or coronary artery disease significantly influenced the likelihood of having a concordant parent or sibling. The likelihood of having an affected sibling increased with the sibship size (OR 1.30 [95%CI 1.14–1.47]). However, no changes were found if the relation between sibling history of stroke and proband characteristics was adjusted for sibship size (table 4).

**Table 4:** Relationship of proband characteristics to family history of stroke in parents and siblings.

Proband characteristics	FHstroke in parents	FHstroke in siblings	
		unadjusted	adjusted for sibship size
**Age**	0.97 (0.95–1.00)*	1.02 (1.00–1.04)	1.02 (0.98–1.05)
**Gender**	0.90 (0.50–1.65)	0.67 (0.34–1.33)	0.77 (0.37–1.57)
**Hypertension**	2.29 (1.21–4.32)*	1.13 (0.56–2.28)	1.11 (0.53–2.32)
**Diabetes mellitus**	2.08 (0.88–4.94)	0.24 (0.55–1.08)	0.33 (0.74–1.50)
**Coronary artery disease**	1.26 (0.52–3.01)	0.77 (0.27–2.18)	0.76 (0.26–2.26)
**Smoking**	0.76 (0.42–1.40)	1.31 (0.65–2.64)	1.23 (0.59–2.56)

Data are depicted as OR (95%CI). FHstroke indicates family history of stroke.

*p<0.05

## Discussion

In FDR of well-subtyped first-ever lacunar stroke patients, we found an almost three-fold increase in GRR of stroke. The increase was more pronounced in patients' parents than siblings. Part of the impressive GRR of 4.52 in parents will be explained by the higher age and the fact that the self-reported prevalence of stroke increases from 2.5% for the ages 55–64 years to 11.6% for those aged over 80 (for males, for females respectively 1.6% to 10.5%).[9] The data on siblings are in line with the average GRR of many complex polygenic diseases,[4] however higher than the figure for overall stroke.[5] Secondly, age of proband and proband status for hypertension influenced the chance of having a parent with a history of stroke and the likelihood of having an concordant sibling increased with sibship size.

The finding of familial aggregation of stroke in lacunar stroke patients has been reported by others.[1], [2], [3] However, these were family history studies in which a positive family history was defined as the presence of at least one affected FDR. The advantage of the GRR above the family history design is that the number of affected family members and the number of known family members is taken into account, providing more information of the magnitude of familial aggregation of stroke.

Besides the advantage of determing the GGR above the FH design, the second strength of our study is the selection of a substantial well subtyped population. A thorough definition of the phenotype is essential before exploring what genes contribute to a disease. We defined a strict phenotype of lacunar stroke, first by combining established criteria of the classical lacunar syndromes with imaging criteria, and second by the exclusion of lacunar stroke patients with a possible cardio- or carotid-embolic stroke cause.[10] Final, we did not classify lacunar stroke based on the presence of risk factors (like hypertension or diabetes), but purely on clinical and radiological criteria, a definition which is suggested for studies on the pathophysiology of lacunar stroke.[11] By this method, we selected patients who developed the stroke most probably due to an intrinsic disease of the cerebral small vessels, and thereby raising the chance of finding genetic factors. Results of recent large genome wide association studies (GWAS) are disappointing which might be explained by small sample sizes and the inclusion of different stroke phenotypes.[12] For future studies, collaboration of several dedicated medical centers is needed as huge numbers of patients need to be included. But also, if stringent uniform classification of lacunar stroke is applied, the chance of finding genes will increase and it will probably limit the number of patients needed.[13]

### Limitations

First, data on subtype of stroke in FDR could not be provided, as we used self-reported history of stroke. Arguments for similar subtypes of stroke in family-members can be found in the literature, for example high heritability of white matter lesions – one of the features of lacunar stroke - in the Framingham Heart Study,[14] more concordant monozygotic than dizygotic twins for white matter lesions,[15] and high prevalence of micro-angiopathic lesions in siblings of lacunar stroke patients.[16] However, we acknowledge that at least some surplus of the stroke in FDR will have different etiology, e.g. cardio-embolic stroke or large-artery stroke. Second, alternative explanations for the aggregation of stroke, besides genetic factors causing stroke, in families of lacunar stroke patients are possible. Shared environmental factors, e.g. diet or smoking habits, or shared intermediate phenotypes, e.g. hypertension, could be responsible for the familial aggregation.[17], [18] We found that proband status of hypertension was related to a parental history of stroke, but as we were unable to collect information about the risk factor profile in FDR of lacunar stroke patients, we cannot provide information of hypertension status in parents. Final, prevalence data on stroke in our direct geographic region were only available in a group FDR of 49 healthy controls. Fortunately, nearby (200 km road-distance) population-based figures were available from the Rotterdam study [9], which we used. As we found a GRR of 0.97 for our controls, we assume that the population in our geographic region is genetically similar to the population of Rotterdam. As the mean age of the Rotterdam population was slightly older than our FDR of lacunar stroke patients, we will not overestimate the risk. Furthermore, the stroke prevalence in FDR of our healthy controls and in the Rotterdam cohort is in line with self reported prevalence's in larger population based cohorts from other European countries, therefore these figures seem quite robust.[19], [20] The composition of our families is similar to other populations, as the median number of sibs in our study was 3, in line with the 954 siblings of 310 probands reported by Meschia [21] and a mean of 3.1 sibling reported by Hassan et al.[22].

### Conclusion

We found an increased GRR of stroke in FDR of patients with lacunar stroke using a classification based on established clinical criteria of lacunar syndromes combined with imaging-findings, without inclusion of a risk-factor profile in the classification system. Selection of such a well defined high risk population for stroke can be a start for further genomic research.

## References

[1] Jood K, Ladenvall C, Rosengren A, Blomstrand C, Jern C (2005). Family history in ischemic stroke before 70 years of age: the Sahlgrenska Academy Study on Ischemic Stroke.. Stroke.

[2] Jerrard-Dunne P, Cloud G, Hassan A, Markus HS (2003). Evaluating the genetic component of ischemic stroke subtypes: a family history study.. Stroke.

[3] Polychronopoulos P, Gioldasis G, Ellul J, Metallinos IC, Lekka NP (2002). Family history of stroke in stroke types and subtypes.. J Neurol Sci.

[4] Burton PR, Tobin MD, Hopper JL (2005). Key concepts in genetic epidemiology.. Lancet.

[5] Lisabeth LD, Peyser PA, Long JC, Majerisk JJ, Smith MA (2008). Stroke among siblings in a biethnic community.. Neuroepidemiology.

[6] Knottnerus IL, Govers-Riemslag JW, Hamulyak K, Rouhl RP, Staals J (2010). Endothelial activation in lacunar stroke subtypes.. Stroke.

[7] Bamford J, Sandercock P, Jones L, Warlow C (1987). The natural history of lacunar infarction: the Oxfordshire Community Stroke Project.. Stroke.

[8] Rouhl RP, van Oostenbrugge RJ, Knottnerus IL, Staals JE, Lodder J (2008). Virchow-Robin spaces relate to cerebral small vessel disease severity.. J Neurol.

[9] Bots ML, Looman SJ, Koudstaal PJ, Hofman A, Hoes AW (1996). Prevalence of stroke in the general population. The Rotterdam Study.. Stroke.

[10] Staals J, van Oostenbrugge RJ, Knottnerus IL, Rouhl RP, Henskens LH (2009). Brain microbleeds relate to higher ambulatory blood pressure levels in first-ever lacunar stroke patients.. Stroke.

[11] Jackson C, Sudlow C (2005). Are lacunar strokes really different? A systematic review of differences in risk factor profiles between lacunar and nonlacunar infarcts.. Stroke.

[12] Markus HS (2010). Unravelling the genetics of ischaemic stroke.. PLoS Med.

[13] Mullen SA, Crompton DE, Carney PW, Helbig I, Berkovic SF (2009). A neurologist's guide to genome-wide association studies.. Neurology.

[14] Atwood LD, Wolf PA, Heard-Costa NL, Massaro JM, Beiser A (2004). Genetic variation in white matter hyperintensity volume in the Framingham Study.. Stroke.

[15] Carmelli D, DeCarli C, Swan GE, Jack LM, Reed T (1998). Evidence for genetic variance in white matter hyperintensity volume in normal elderly male twins.. Stroke.

[16] Leistner S, Huebner N, Faulstich A, Ludwig D, Rees M (2008). Increased prevalence of microangiopathic brain lesions among siblings of patients with lacunar stroke. A prospective multicenter study.. Eur Neurol.

[17] Flossmann E, Schulz UG, Rothwell PM (2005). Potential confounding by intermediate phenotypes in studies of the genetics of ischaemic stroke.. Cerebrovasc Dis.

[18] Lindgren A, Lovkvist H, Hallstrom B, Hoglund P, Jonsson AC (2005). Prevalence of stroke and vascular risk factors among first-degree relatives of stroke patients and control subjects. A prospective consecutive study.. Cerebrovasc Dis.

[19] Jungehulsing GJ, Muller-Nordhorn J, Nolte CH, Roll S, Rossnagel K (2008). Prevalence of stroke and stroke symptoms: a population-based survey of 28,090 participants.. Neuroepidemiology.

[20] Geddes JM, Fear J, Tennant A, Pickering A, Hillman M (1996). Prevalence of self reported stroke in a population in northern England.. J Epidemiol Community Health.

[21] Meschia JF, Brown RD, Brott TG, Hardy J, Atkinson EJ (2001). Feasibility of an affected sibling pair study in ischemic stroke: results of a 2-center family history registry.. Stroke.

[22] Hassan A, Sham PC, Markus HS (2002). Planning genetic studies in human stroke: sample size estimates based on family history data.. Neurology.

